# Precision medicine^☆^

**DOI:** 10.1016/j.bjorl.2018.02.002

**Published:** 2018-02-24

**Authors:** Marcio Nakanishi

**Affiliations:** Universidade de Brasília, Faculdade de Medicina da Universidade de Brasília (FM-UnB), Departamento de Cirurgia – Programa de Pós-Graduação em Ciências Médicas, Brasília, DF, Brazil

Precision Medicine has been a growing topic of discussion in the last decade. More than 17,000 articles have been published on this subject only in the last 5 years. In 2015, the US National Institutes of Health launched the Precision Medicine Initiative, which defines it as “an emerging approach to disease prevention and treatment that takes into account the individual variability in genes, environment, and lifestyle for each person”.^1^

The concept of precision medicine is not new. Accurate diagnosis and specific treatment have always been the target of health systems. For instance, ABO/Rh blood typing, used since 1940,^2^ allows the identification of individual variability and, consequently, an adequate transfusion for the specific blood group. Overall, Precision Medicine is on the opposite side of the “one size fits all” concept, in which a single type of disease treatment is developed for an average patient, not taking into account individual differences.^1^, ^3^

The term “Personalized Medicine” is used interchangeably with the term “Precision Medicine”. However, about this specific taxonomy, the National Research Council explains: “Precision Medicine” refers to medical treatment adapted to the individual characteristics of each patient. It does not literally mean the creation of unique drugs or medical devices for a patient, but rather the ability to classify individuals into subpopulations that differ in their susceptibility to a particular disease, in the biology and/or prognosis of those diseases or in their response to a specific treatment. Preventive or therapeutic interventions can then focus on the ones that will benefit, saving costs and side effects for those who will not. Even though the term “Personalized Medicine” is also used to convey this meaning, this term is sometimes misinterpreted as implying that unique treatments can be designed for each individual. For this reason, the committee considers the term “Precision Medicine” as preferable to “Personalized Medicine”.^4^

Precision Medicine advances in parallel to the evolution of biological-molecular measuring instruments and analytical informatics. While molecular biology quantifies and characterizes the variability of genes, proteins and metabolites, the analysis of these voluminous and complex data, in the field called big data analytics, seeks to predict the behavior of diseases and individuals, thus generating the possibility of preventive interventions and “personalized” treatment.

Cystic fibrosis, a genetic disease that modifies the cell membrane receptor that controls ionic transport in sweat, digestive juice and mucus, both nasal and pulmonary, affects mainly the skin, digestive and respiratory systems. Its diagnosis and treatment are an example in the field of Precision Medicine. The disease is caused by a mutation in chromosome 7,^5^ and drugs such as Ivacaftor and Lumacaftor are specific for each type of mutation. A specific genetic diagnosis allows the precise choice of the drug and, consequently, better therapeutic results.^6^

The use of mathematical algorithms and artificial intelligence systems that take into account individual genetic and phenotypic variability, associated with the environment and lifestyle, opens up a perspective for the use of a precision diagnosis at a population scale. The accurate diagnostic prediction, as well as the causal factors involved, will allow the design of preventive measures and specific treatments for each type of patient, in a precise and personalized manner.

## Conflicts of interest

The author declares no conflicts of interest.
